# To scavenge or not to scavenge, that is STILL the question

**DOI:** 10.1107/S0909049512046237

**Published:** 2012-12-05

**Authors:** Elizabeth G. Allan, Melissa C. Kander, Ian Carmichael, Elspeth F. Garman

**Affiliations:** a Laboratory of Molecular Biophysics, Department of Biochemistry, University of Oxford, South Parks Road, Oxford OX1 3QU, UK; b Notre Dame Radiation Laboratory, and Department of Chemistry and Biochemistry, University of Notre Dame, IN 46556, USA

**Keywords:** radiation damage, crystallization buffers, radicals, scavengers, lithium nitrate

## Abstract

An extensive radiation chemistry literature would suggest that the addition of certain radical scavengers might mitigate the effects of radiation damage during protein crystallography diffraction data collection. However, attempts to demonstrate and quantify such an amelioration and its dose dependence have not yielded consistent results, either at room temperature (RT) or 100 K. Here the information thus far available is summarized and reasons for this lack of quantitative success are identified. Firstly, several different metrics have been used to monitor and quantify the rate of damage, and, as shown here, these can give results which are in conflict regarding scavenger efficacy. In addition, significant variation in results from data collected from crystals treated in nominally the same way has been observed. Secondly, typical crystallization conditions contain substantial concentrations of chemical species which already interact strongly with some of the X-ray-induced radicals that the added scavengers are intended to intercept. These interactions are probed here by the complementary technique of on-line microspectrophotometry carried out on solutions and crystals held both at 100 K and RT, the latter enabled by the use of a beamline-mounted humidifying device. With the help of computational chemistry, attempts are made to assign some of the characteristic spectral features observed experimentally. A further source of uncertainty undoubtedly lies in the challenge of reliably measuring the parameters necessary for the accurate calculation of the absorbed dose (*e.g.* crystal size and shape, beam profile) and its distribution within the volume of the crystal (an issue addressed in detail in another article in this issue). While microspectrophotometry reveals that the production of various species can be quenched by the addition of scavengers, it is less clear that this observation can be translated into a significant gain in crystal dose tolerance for macromolecular crystallographers.

## Abstract 2

The reasons for the conflicting results on the efficacy of scavengers in macromolecular crystallography are examined in the light of some new results.

## 1. Introduction

With the ever-increasing X-ray photon fluxes now available at third-generation synchrotrons, radiation damage inflicted on experimental samples has once again become a concern during macromolecular crystallographic (MX) investigations, leading to failure of a significant number of structure solution attempts. As is now well documented and has been reviewed (Garman, 2010 ▶; Holton, 2009 ▶), two types of damage are broadly observed. Global damage, perhaps best quantified by the decline in the integrated intensity of the diffraction patterns as data collection proceeds, is apparently governed by processes of a physical nature. It is thought that there is an upper limit to the dose that can be tolerated by a biological crystal, calculated by analogy with observations of radiation damage rates in electron crystallography to be 20 MGy (Gy = J kg^−1^) (Henderson, 1990 ▶). An experimental dose limit at 100 K has been determined as 43 MGy, the dose that will halve the initial summed diffraction intensity across all resolution bins, and a maximal value of 30 MGy has been proposed, beyond which biological information is likely to be compromised (Owen *et al.*, 2006 ▶).

However, damage to certain specific residues, a chemical phenomenon, is often observed at much lower doses. This specific damage has been reported to occur in a characteristic sequence in a number of metal-free crystals. Disulfide bridges are the first to be impacted, followed by the decarboxylation of aspartate and glutamate residues, loss of the OH group from tyrosines and the scission of the C—S bond in methionines (Burmeister, 2000 ▶; Ravelli & McSweeney, 2000 ▶; Weik *et al.*, 2000 ▶). At 100 K, the only mobile species are presumably electrons (Jones *et al.*, 1987 ▶) and positive holes since 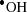 radicals are widely thought to be immobile below 110 K. Recently published results of experiments in which an absorption peak at 240 nm from an irradiated vitrified crystallization solution was monitored with a microspectro­photometer and postulated to be from 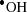 (produced from holes in the aqueous solvent) suggests that these radicals are not able to diffuse and recombine until the temperature is raised to around 160 K (Owen *et al.*, 2012 ▶). Thus the radiation chemistry mechanisms of specific damage at 100 K are most likely confined to pathways instigated by holes (either in or adjacent to the protein) and mobile electrons. Of course, in media of high acidity these electrons will react rapidly with protons to give hydrogen atoms which are presumably also mobile at cryotemperatures (Shiraishi *et al.*, 1976 ▶).

Various efforts have been made to reduce the rate of radiation damage and thus enable useful data collection over longer beam exposures. Cryocooling (Garman & Schneider, 1997 ▶) has been reported to offer a damage reduction of approximately a factor of 70 compared with room temperature (RT) studies (Nave & Garman, 2005 ▶). However, certain crystals are not suitable for cryogenic studies, such as those of particular viruses, and data can usually only be collected for these samples at RT. Also, *in situ* data collection from crystallization trays is increasingly being used at synchrotron sources and general interest in RT protein crystallography is re-emerging as doubts arise about the conformational bias of protein ensembles that flash cooling may introduce (Fraser *et al.*, 2011 ▶). As a result, there is renewed interest in finding innovative and practical solutions to the problem of radiation damage in protein crystallography at both cryogenic temperatures and RT. One promising avenue of research is the use of radical scavengers, intended to intercept damage agents.

Over the last decade, conflicting results have been reported as to the efficacy (or otherwise) of various radical scavengers in reducing the rate of radiation damage in RT and 100 K MX (Murray & Garman, 2002 ▶; Kauffmann *et al.*, 2006 ▶; Southworth-Davies & Garman, 2007 ▶; Nowak *et al.*, 2009 ▶; Barker *et al.*, 2009 ▶; Macedo *et al.*, 2009 ▶; De la Mora *et al.*, 2011 ▶; Kmetko *et al.*, 2011 ▶). Several different metrics have been used to monitor and quantify the rate of damage, including the loss of diffraction intensity summed over all resolution bins of complete datasets normalized to the total summed intensity of the first data set (*I*_*n*_/*I*_0_), the difference in intensity of the same reflection (*R*_d_) measured at different times (and thus different dose) in the data collection (Diederichs, 2006 ▶), and the difference in scaling *B*-factor between consecutive data sets or between repeated 5°-wedges, *B*_rel_ (Kmetko *et al.*, 2006 ▶). It has already been noted that for *I*_*n*_/*I*_0_ (De la Mora *et al.*, 2011 ▶), and for *B*_rel_ and *R*_d_ (Krojer & von Delft, 2011 ▶) calculations using different software packages can give different values and thus change the conclusions that can be drawn from the rates of global radiation damage obtained for the same raw data series. Other metrics of global radiation damage include unit cell expansion and crystal mosaicity. However, these appear to be poorly reproducible (Murray & Garman, 2002 ▶; Ravelli *et al.*, 2002 ▶). It should be noted that the ratio of average intensity of reflections to noise, *I*/σ(*I*), is an inherently biased metric since noise increases with radiation damage (De la Mora *et al.*, 2011 ▶).

Significant variation between crystals treated in nominally the same way with scavengers has been observed, with a large scatter in the results. In addition, some scavengers have been tested by several groups and the results are not always in accord. Indeed a number of studies have observed little, or even adverse effects of these additives. For the cases where results from investigations by different researchers conflict, the metrics employed in each study and the resulting conclusions are summarized in Table 1 ▶ (a full summary of all scavengers so far tried for MX can be found in the supplementary material, Table S1^1^). It can be seen from examination of Table 1 ▶ that only two scavengers have been reported to increase the dose tolerance by more than a factor of two, and that there is a lack of consensus on the efficacy of those that have been investigated by various different means. The exceptions are 1,4-benzoquinone at RT and sodium nitrate at 100 K. The former has been observed to increase the dose tolerance to global damage by a factor of nine (Barker *et al.*, 2009 ▶) as monitored by intensity decay, and significantly reduce the specific damage to susceptible residues (with some disulfides remaining undamaged). In the latter case the specific damage to disulfides was reduced by more than a factor of five (De la Mora *et al.*, 2011 ▶), while the global damage, again assessed from intensity decay, was lessened by a factor of two. Both of these scavengers were tested on chicken egg-white lysozyme (HEWL) crystals.

Here, possible reasons for these ambiguous and inconsistent observations (Table 1 ▶) are identified, analysed and investigated.

Firstly, when assessing global damage, the same diffraction data analysed using the various damage metrics are shown to provide conflicting results as to the crystal dose tolerance, resulting in differing conclusions on scavenger efficacies.

Secondly, the radiation chemistry already taking place in the crystallization buffers upon X-irradiation is shown to strongly compete with any attempts to quench damage agents with added scavengers. The results of complementary microspectrophotometric studies are presented both at cryo- and room temperatures on mounted crystals, the crystallization screens, and also their individual components. It is clear that there is hitherto unexplored interplay between the components in crystallization buffers. This phenomenon greatly affects the reproducibility of the results and offers a further explanation for some of the above-mentioned discrepancies.

Computational chemistry investigations have also been pursued in an effort to shed light on the observed absorption patterns and hence elucidate the fate of damage agents induced in the various supporting media.

Thirdly, it should be noted that the accurate determination of the dose which a crystal has absorbed is a decidedly non-trivial exercise. An estimation of the dose requires detailed information on the characteristics of both the crystal and the beam. For the crystal, the absorption coefficient is determined from knowledge of the sample size and composition (*i.e.* the number of each atom type in the unit cell), and for the incident beam, its energy, size, shape and flux (in photons s^–1^) must be known. For MX, this can be conveniently carried out by means of the program *RADDOSE* (Murray *et al.*, 2004 ▶; Paithankar *et al.*, 2009 ▶). Detailed considerations of dose and the effects of its distribution are explored elsewhere in this issue (Zeldin *et al.*, 2013 ▶).

In addition to these factors, the whole concept of a dose limit based on the arguments of physics is challenged if dose tolerance can be chemically modified by scavengers. Thus it may be time to consider rethinking the current division of effects into ‘physical’ and ‘chemical’. This important question will be revisited in the discussion.

## 2. Methods

### 2.1. Re-analysis of 100 K HEWL–sodium-nitrate and HEWL–ascorbate scavenger data using different metrics for global radiation damage

To test the robustness of global damage metrics as a source of the inconsistent scavenger results from different studies, the raw data for which *I*/*I*_0_ [obtained from both *SCALA* (Evans, 2006 ▶) and *XDS/XSCALE* (Kabsch, 2010 ▶)] and *R*_d_ analyses of full datasets were reported by De la Mora *et al.* (2011 ▶) were reanalysed. Using *MOSFLM*, *SCALA*, *CAD* and *SCALEIT* from *CCP4i* (Winn *et al.*, 2011 ▶), values were produced for *I*/*I*_0_ (*SCALA*) and *B*_rel_ (*SCALEIT*) both from full data sets and from 5° wedges of data at various doses.

The original diffraction data were acquired at the ESRF at 100 K from the samples shown in Table 2 ▶ (De la Mora *et al.*, 2011 ▶). For the HEWL crystals soaked in 1 *M* sodium nitrate, this scavenger was clearly shown to be effective in supressing specific damage by means of electron density maps, and both ascorbate and nitrate increased the dose tolerance to global damage by around a factor of two.

The HEWL crystals used in the De la Mora *et al.* study were grown using 50 mg ml^−1^ HEWL protein (from Sigma) in 200 m*M* sodium acetate (NaAc) buffer at pH 4.7 mixed with an equal volume of precipitant consisting of 0.2 *M* NaAc buffer at pH 4.7 containing 10% *w*/*v* NaCl. Cryoprotection was achieved by replacing the water in the original precipitant solution by 30% glycerol (*v*/*v*) and soaking the crystals in this for approximately 1 min. HEWL–ascorbate co-crystals were grown by replacing water in the precipitate solution to give a final concentration of 1 *M* sodium ascorbate. The sodium nitrate treated crystals were soaked for 4 min or 8 min in a cryosolution made up as above but with some of the water replaced by scavenger to give a final concentration of 0.5 *M* nitrate.

### 2.2. Microspectrophotometry measurements

All microspectrophotometery was performed at the Diamond Light Source. Samples were studied using the same on-line microspectrophotometer (R. L. Owen, private communication), but two different beamlines having different beam profiles were used: I02 with horizontal (H) and vertical (V) FWHM of 110 µm and 70 µm, respectively, at 12.658 keV giving 3 × 10^12^ photons s^−1^ at full transmission, and I24 with FWHMs of either 30 µm (H) and 30 µm (V) or 50 µm (H) and 50 µm (V) at 12.8 keV giving 1 × 10^12^ photons s^−1^ at full transmission.

The on-line off-axis UV–vis microspectrophotometer consists of mirror lenses (Bruker), a halogen/deuterium lamp (Ocean Optics) and two objective lenses mounted at 45° with respect to the X-ray beam, allowing cryostream access. The microspectrophotometer is fitted with a Shamrock 303 imaging spectrograph (Andor) which has a wavelength detection range of 200–760 nm. The dimensions of the light beam at the crystal were 80 µm × 80 µm and the device was fitted with 400 µm- and 200 µm-diameter incoming and outgoing optic fibres, respectively. Spectra were collected over an exposure time of 30 ms with 25 accumulations per spectrum written to disk (*i.e.* 0.75 s per spectrum).

Crystals of HEWL were grown using the same components at slightly different concentrations as those used in the De la Mora *et al.* study: 50 mg ml^−1^ HEWL in 0.1 *M* NaAc at pH 4.8 was mixed with an equal volume 0.1 *M* NaAc at pH 4.8, 1.4 *M* NaCl. Crystals grew in a variety of sizes with cell dimensions of *a* = *b* = 77.78 Å and *c* = 38.45 Å and α = β = γ = 90° in space group *P*4_3_2_1_2. Cubic bovine pancreatic insulin crystals were grown using a lyophilized powder (Sigma I6634) from 20 mg ml^−1^ protein in 0.02 *M* Na_2_HPO_4_, pH 10.4, and 0.01 *M* Na_3_EDTA, mixed with 0.5 *M* Na_2_HPO_4_/Na_3_PO_4_, pH 10.4. Crystals of between 15 × 15 × 15 µm and 70 × 70 × 70 µm grew within 72 h with cell dimension of *a* = 78.9 Å in space group *I*2_1_3.

Scavenger (1 *M* LiNO_3_ or NaNO_3_: final concentration 0.5 *M*) was introduced into the crystals by soaking for 4 min. Both the crystal and solution samples were mounted using normal cryoloops (rayon) open to the air. Initial tests showed that the PET tube usually used in the MiTeGen RT mounting system (Kalinin *et al.*, 2005 ▶) to enclose the crystal and prevent dehydration was inappropriate as it caused scattering in the absorption spectra. Therefore, an HC1b humidity-controlling device (Sanchez-Weatherby *et al.*, 2009 ▶) was used to prevent dehydration of samples at RT. Samples tested at cryogenic temperatures, which had been cryoprotected (by replacing the water in the original precipitant solution by 30% *v*/*v* glycerol), were held across rayon cryoloops and flash cooled into 100 K gaseous nitrogen.

Absorption spectra were collected using two different protocols: with beam on continuously for 10 or 20 s or with beam on for 2 s and then off for 3 s, the cycle being repeated ten times. Data were analysed using the Andor *SOLIS* software supplied with the microspectrophotometer.

Doses were calculated using *RADDOSE* (version 2) following calibration of each beamline with a 500 µm-thick silicon diode (Hamamatsu) by the method detailed by Owen *et al.* (2009 ▶).

### 2.3. Computational chemistry

In an effort to identify the sources of the absorption peaks detected by microspectrophotometry during X-irradiation, extensive computational chemistry investigations^2^ on the individual components and their mixtures were conducted.

The structures of the initial compounds and their various radiolytic products, and intermediates along the path to their production, were determined by density functional theory (DFT) calculations. The standard B3LYP functional (Becke, 1993 ▶) was employed with a compact polarized split-valence basis set, denoted 6-31G*. Geometries were optimized using analytic gradients and the nature of the various stationary points determined by consideration of the analytic Hessian. Ultrafine integration grids were used throughout. The effect of the surrounding environment was modelled using a simple reaction field treatment within the integral equation formalism of the polarized cavity approach (IEFPCM) (Tomasi *et al.*, 1999 ▶). The geometries of a subset (lowest energy conformers) of the obtained structures were then further refined using a more flexible 6-311+G(d,p) basis set which also includes diffuse functions on the heavy atoms and polarization functions on the hydrogen atoms. The thermochemical feasibility of various reaction paths was assessed and key transient intermediates, possibly observable by other investigative techniques, were identified.

Absorption spectra characteristic of the various intermediates and products were calculated from time-dependent density functional theory (TD-DFT) (Runge & Gross, 1984 ▶) at the corresponding optimized geometries of the species involved, again using a diffuse function augmented (6-31+G*) basis set and within the same solvent reaction field model.

## 3. Results

### 3.1. Re-analysis of HEWL–sodium-nitrate scavenger data using different metrics for global radiation damage

**Figure 1:**
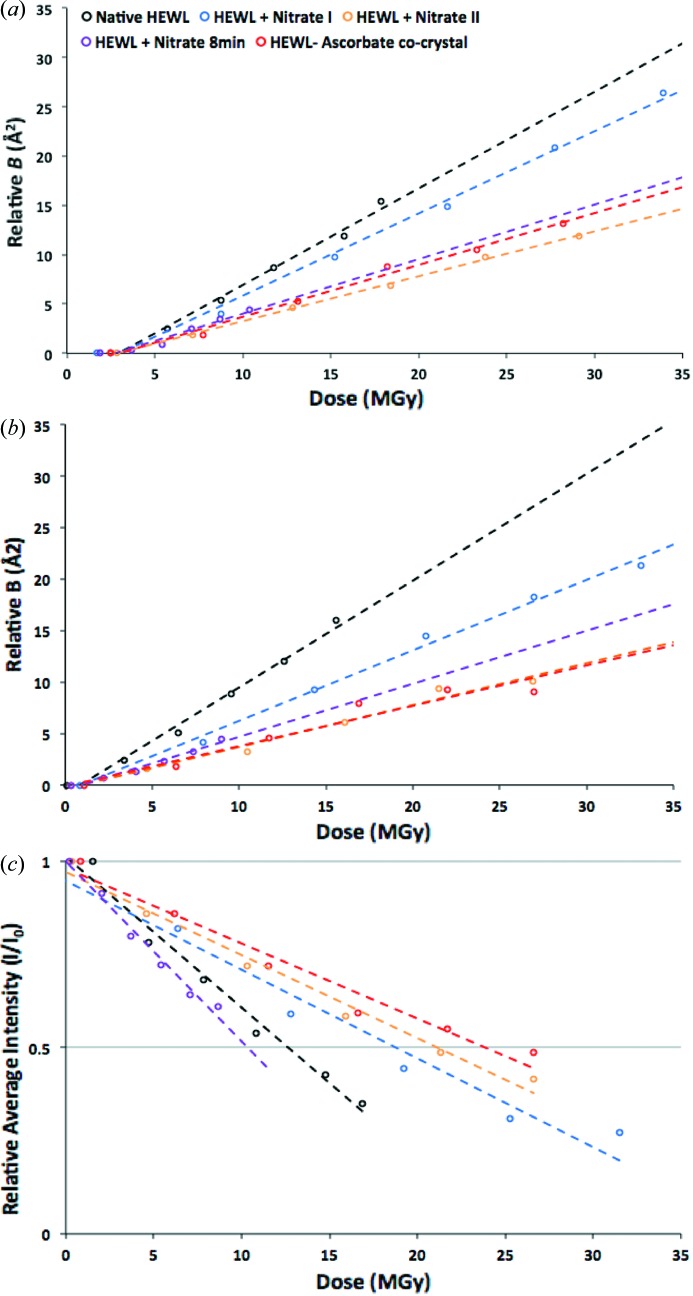
Results of a reanalysis of the data reported by De la Mora *et al.* (2011 ▶). (*a*) *B*_rel_ for whole data sets and (*b*) for 5° wedges, and (*c*) the *I*/*I*_0_ analysis.

The results of the reanalysis of the previously published HEWL–sodium-nitrate data to extract *B*_rel_ for whole data sets (Fig. 1*a* ▶) and for 5° wedges (Fig. 1*b* ▶) implied different efficacy of sodium nitrate as a scavenger than was obtained from the original *I*/*I*_0_ analysis (Fig. 1*c* ▶). From these graphs it is clear that there is a disparity between the metrics. Generally, there is a consistent trend in the results, but the HEWL crystal soaked in nitrate for 8 min can be described as being either less prone or more prone to global radiation damage depending on whether *B*_rel_ or *I*/*I*_0_ is used as the metric, respectively. The results also show that *B*_rel_ derived from 5° wedges of data (*B*_rel_5) does not give exactly the same description of global damage as *B*_rel_ derived from whole datasets (*B*_rel_DS). The general trend is again conserved, but there are notable differences in the suggested extent to which crystal samples are protected from global radiation damage compared with the native crystal. For example, comparisons of the global radiation damage in the ascorbate co-crystal with the native crystal show greater protection when using *B*_rel_5 as a metric as compared with using *B*_rel_DS. Previous *R*_d_ analysis of these data (De la Mora *et al.*, 2011 ▶) showed ascorbate to give a 2.5-fold lower increase in decay factor after 20 MGy than that observed in the native crystal. Interestingly, at this dose, agreement is found with the *B*_rel_5 analysis, which shows a similar level of protection at 20 MGy. The values of *D*_1/2_ and the Δ*B*_rel_/Δ*D* resulting from the analyses are given in Table 3(*a*) ▶.

The intensity (*I*/*I*_0_) decay data can be compared by defining an enhancement factor, *E*_I_, for the intensity analysed data as the *D*_1/2_ (dose required to halve the intensity of the dataset) value for each sample divided by the *D*_1/2_ value for the native HEWL crystal. Thus *E*_I_ > 1 suggests protection from global radiation damage, a value of 1 suggests no difference in susceptibility, and *E*_I_ < 1 implies increased sensitivity. For the *B*_rel_ analysis, a smaller change in *B*_rel_ with dose indicates lower damage, so the ratio of the gradients of the native to scavenger fitted *B*_rel_ lines can be used to judge scavenger efficacy (*E*_B_). Table 3(*b*) ▶ highlights the differences in the extent to which the crystal samples including scavenger are protected from global radiation damage compared with the native crystal, suggested by the different metrics. As can be seen from that table, the *I*/*I*_0_ metric orders the scavengers’ effectiveness as ascorbate > nitrate II > nitrate I > nitrate8, whereas *B*_rel_5 gives ascorbate > nitrate II > nitrate8 > nitrate I, while *B*_rel_DS gives nitrate II > ascorbate > nitrate8 > nitrate I.

### 3.2. Microspectrophotometry at RT and 100 K

**Figure 2:**
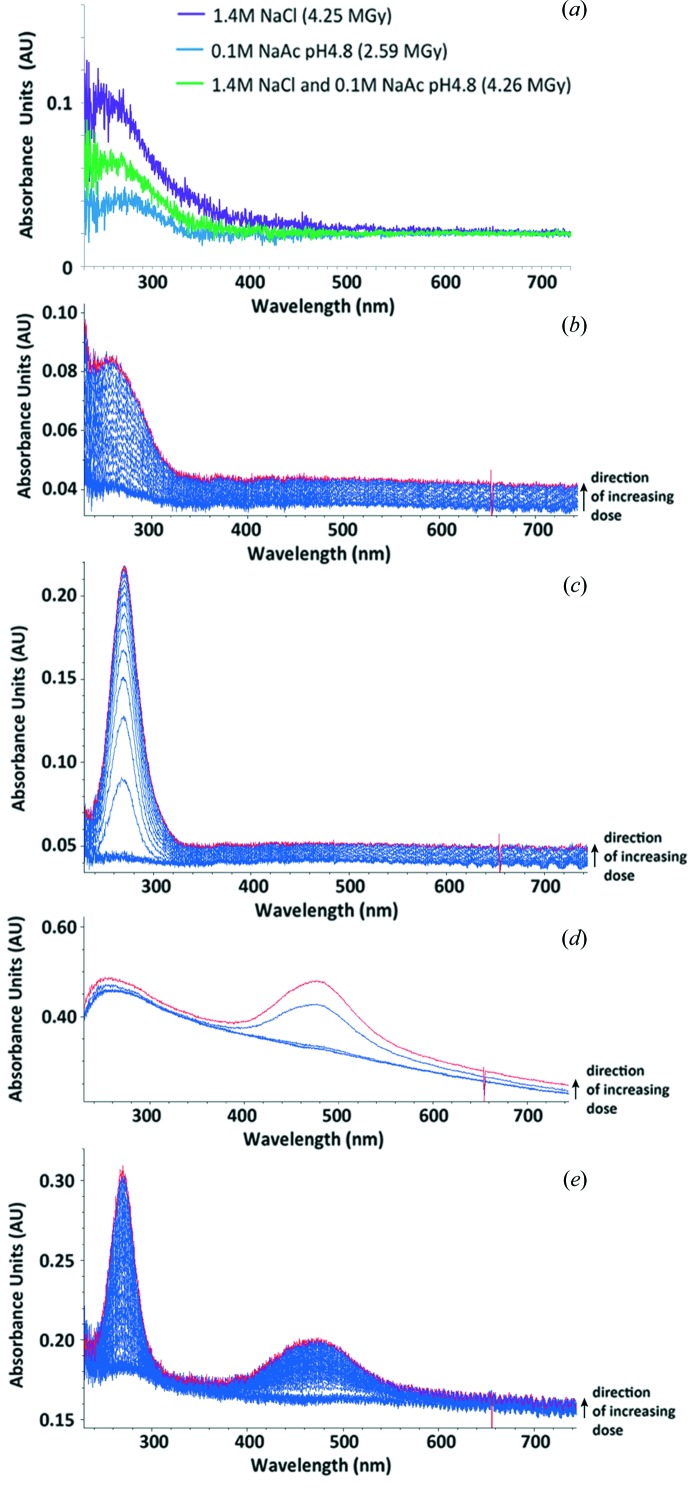
On-line microspectrophotometric analysis of HEWL crystallization buffer and its components at RT. (*a*) A difference absorption spectrum (spectra after irradiation − spectra before irradiation) of the HEWL buffer constituents in water (purple: 1.4 *M* NaCl at 4.25 MGy; green: 0.1 *M* NaAc at 2.59 MGy; blue: both at 4.26 MGy). (*b*) A stacked absorbance spectrum of 30% glycerol upon irradiation, to a final dose of 0.27 MGy as indicated by the red plot. (*c*) A stacked absorbance spectrum of 30% glycerol and 0.1 *M* sodium acetate pH 4.8, upon irradiation, to a final dose of 0.3 MGy as indicated by the red plot. (*d*) A stacked absorbance spectrum of 30% glycerol and 1.4 *M* sodium chloride upon irradiation, to a final dose of 0.14 MGy as indicated by the red plot. (*e*) A stacked absorbance spectrum of the crystallization buffer (30% glycerol, 0.1 *M* sodium acetate pH 4.8, 1.4 *M* sodium chloride) upon irradiation, to a final dose of 0.39 as indicated by the red plot.

**Figure 3:**
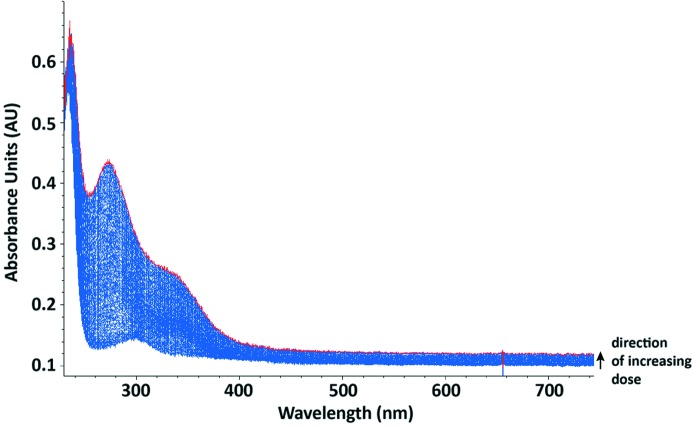
A stacked absorption spectrum of 0.5 *M* sodium nitrate in HEWL cryobuffer (crystallization buffer with water replaced by 30% glycerol) upon irradiation, to a final dose of 0.40 MGy as indicated by the red plot.

**Figure 4:**
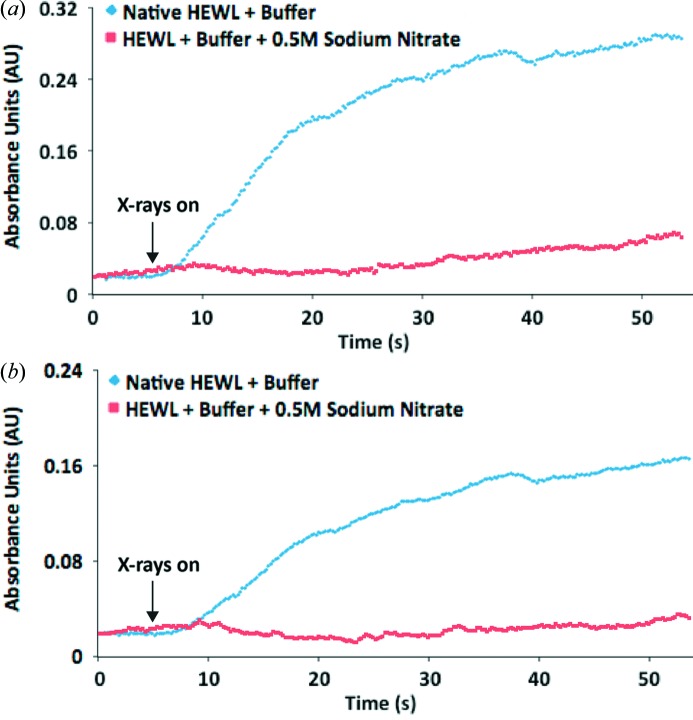
Changes in absorbance at (*a*) 400 nm, (*b*) 580 nm occurring upon irradiation of 35 mg ml^−1^ HEWL solutions, with and without 0.5 *M* sodium nitrate at RT. Samples irradiated to a final dose of 1 MGy.

The availability of a suitably equipped microspectro­photometer allows the detection of optical absorption changes induced by X-ray irradiation of solutions and crystals both at RT and 100 K. In an attempt to decouple parameters affecting this phenomenon, and thus gain insight into the radiation chemistry, various subsets of the components of the total irradiated volume have been analysed independently. In Fig. 2 ▶, the absorption by the cryobuffer, consisting of the mother liquor and glycerol, has been analysed at RT along with those of the separate components and combinations thereof. Fig. 3 ▶ shows the RT spectrum obtained from 0.5 *M* NaNO_3_ in HEWL cryobuffer (without the protein) before and after irradiation, and Fig. 4 presents RT time traces at selected wavelengths (400 nm and 580 nm) from HEWL solution in cryobuffer.

**Figure 5:**
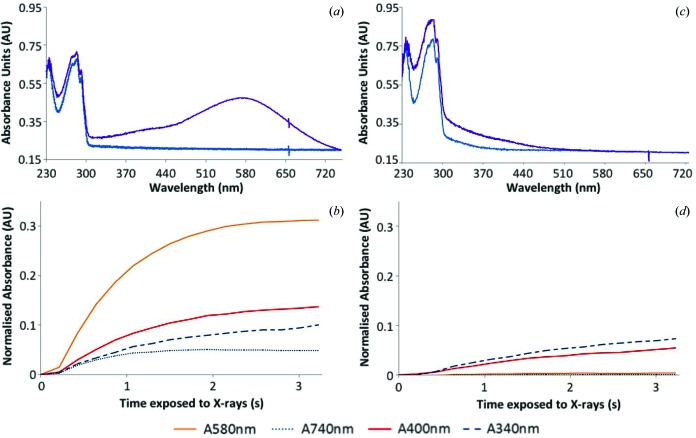
Changes in absorbance at 100 K occurring upon irradiation of 35 mg ml^−1^ HEWL solutions in cryobuffer, with (*c* and *d*) and without (*a* and *b*) 0.5 *M* lithium nitrate. (*a* and *c*) Absorption spectra at a dose of 0 MGy (blue) and 0.078 MGy (purple). (*b* and *d*) Changes in normalized absorbance with time at certain wavelengths of interest. A 1 s X-ray exposure gave an absorbed dose of 0.02 MGy.

**Figure 6:**
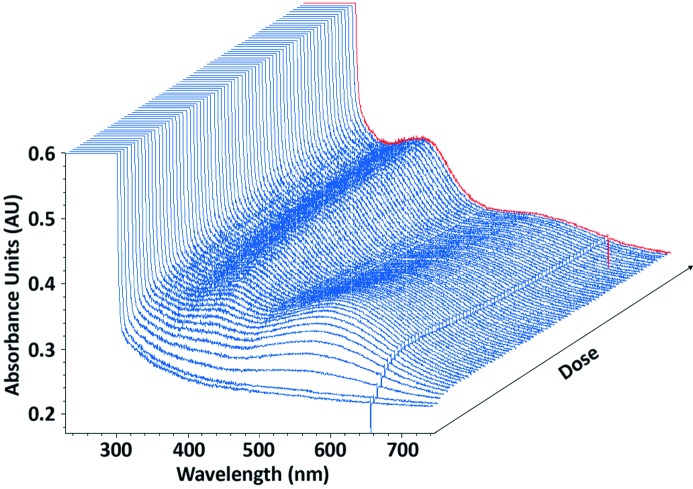
Three-dimensional absorbance spectrum viewed at 45° of a native HEWL crystal irradiated at 100 K to a final dose of 0.26 MGy (red line).

**Figure 7:**
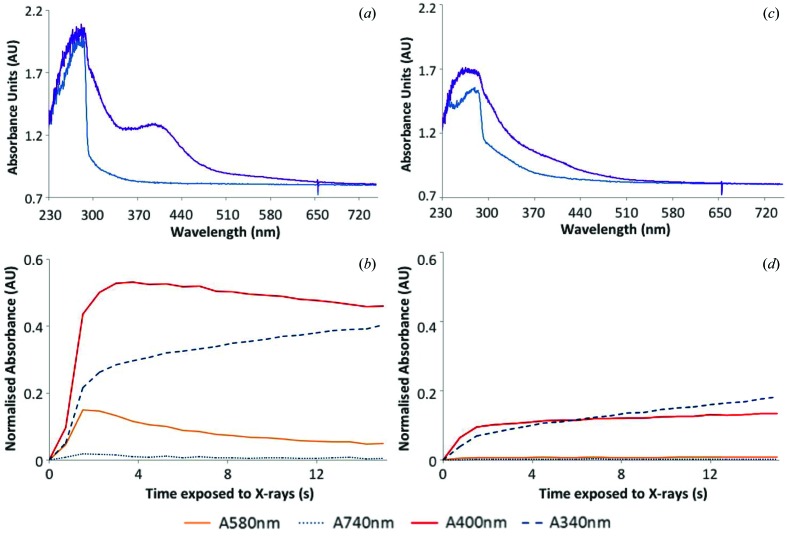
Changes in absorbance at 100 K occurring upon irradiation of a cubic insulin crystal, soaked (*c* and *d*) and not soaked (*a* and *b*) in 0.375 *M* lithium nitrate. (*a* and *c*) Absorption spectra at a dose of 0 MGy (blue) and 2.73 MGy (purple). (*b* and *d*) Changes in normalized absorbance with time at certain wavelengths of interest. 1 s of X-ray exposure is equivalent to a dose of 0.18 MGy.

For HEWL solution at 100 K, the effect of adding scavenger, LiNO_3_, on X-ray-induced spectral changes is demonstrated in Fig. 5, along with time traces at selected wavelengths. Fig. 6 contains time traces at 100 K from irradiated native HEWL crystals. Fig. 7 demonstrates the effect of the addition of an electron scavenger, again LiNO_3_, in insulin crystals at 100 K. The time courses of the absorption are also presented. The observations illustrated in these figures are described in more detail below.

#### 3.2.1. HEWL buffer component analysis

Absorption peaks observed at RT upon X-ray irradiation of the HEWL mother liquor constituents (Fig. 2 ▶) demonstrate that the presence of the cryoprotectant, glycerol, has an effect on the changes in absorbance. At RT the results clearly show that both NaAc and NaCl, alone and in combination, give only a broad low-intensity signal at short wavelength (<300 nm). Glycerol alone yields a much stronger broad absorbance around 255 nm, a principal contributor which can be assigned to the enol of malonic dialdehyde (MDA) based both on TD-DFT calculations in this work and on values from the literature (Ivanova *et al.*, 2009 ▶). However, when glycerol is added to NaAc a clear peak at 270 nm is observed, whereas glycerol with NaCl gives a broader signal at 470 nm.

#### 3.2.2. Investigating the scavenging ability of nitrate at RT

Before testing the RT radical quenching efficacy of nitrate in HEWL, a 0.5 *M* NaNO_3_ solution in HEWL cryoprotected mother liquor (without any protein) was irradiated and the absorbance monitored. Before irradiation, the absorption spectrum showed only two peaks, known to be characteristic of nitrate: an intense signal at 230 nm extending to lower wavelengths and a much weaker one at 300 nm (Ley, 1928 ▶). After irradiation, a 250 nm peak tentatively attributable to NO_3_^2−^ [assignment based on Grätzel *et al.* (1970 ▶)] appeared as a large shoulder on the 270 nm peak previously observed from irradiation of acetate and glycerol, but the 470 nm peak seen in the NaCl and glycerol sample (see §3.2.1) was quenched. A large shoulder extending upwards in wavelength from the 270 nm peak to around 380 nm was recorded, centred at approximately 340 nm (Fig. 3 ▶). Some contribution to this signal is likely to be made by the Cl_2_^–^ radical anion which has a substantial extinction coefficient and an absorption peaking at 340 nm (Jayson *et al.*, 1973 ▶).

RT time courses at the wavelengths characteristic of disulfide radical anions [400 nm (Hoffman & Hayon, 1972 ▶)] and solvated electrons [580 nm (McGeehan *et al.*, 2009 ▶)] from an irradiated 35 mg ml^−1^ HEWL solution in cryoprotected mother liquor are shown in Figs. 4(*a*) and 4(*b*) ▶. Before irradiation, the absorption spectrum (not shown) showed only a clean peak from the protein at 280 nm, but, upon interaction with X-rays, the 250 nm MDA signal develops and a broad low-intensity 580 nm peak attributable to solvated electrons also appeared. In addition, a shoulder from the 280 nm protein peak extending to a wavelength of 500 nm appeared. This can be assigned as a combination of the 340 nm peak mentioned above with potential contributions from cyclohexadienyl and hydroxycyclohexadienyl radicals, formed from hydroxyl radical attack on the aromatic residues in the protein, in addition to the 400 nm signal expected from the disulfide radical anion. It also possibly obscures any contribution to the absorbance at 470 nm observed from the NaCl/glycerol mixture (§3.2.1). Other absorbances noted above for the various combinations of mother liquor components are dwarfed by the intrinsic absorptions due to the protein.

The same solution with 0.5 *M* NaNO_3_ included was then monitored and both the 400 nm and 580 nm peaks were quenched. The time courses of these absorbances with added NaNO_3_ are also displayed in Figs. 4(*a*) and 4(*b*) ▶.

Interestingly, in-house diffraction experiments carried out on a Bruker MicroStar generator and a Mar345 imaging-plate detector on three native and three LiNO_3_-soaked (0.5 *M* for 4 min) HEWL crystals showed no difference between the rate of decrease of *I*/*I*_0_ (*E*_I_ = 0.95) but a slight protecting effect if judged by *B*_rel_5 (ratio of Δ*B*/Δ*D* without and with scavenger, *E*_B_ = 1.3) or *B*_rel_DS (1.2) for the two sets of crystals (data not shown). A protective effect of 2.1 (as judged by the *B*_rel_5 metric) for RT HEWL soaked in 0.1 *M* NaNO_3_ has previously been reported by Kmetko *et al.* (2011 ▶). Our observation that nitrate does not significantly modify global damage rates at RT is explored in §4.

#### 3.2.3. Investigating the scavenging ability of nitrate at 100 K

Irradiation of a native HEWL cryobuffered protein solution at 100 K results in the formation of peaks at 400 nm and 580 nm in the absorption spectrum [Figs. 5(*a*) and 5(*b*) ▶], which are attributed to disulfide anion radicals and solvated electrons, respectively. Figs. 5(*c*) and 5(*d*) ▶ show that addition of 0.5 *M* LiNO_3_ to the protein solution quenches these peaks to the baseline levels of absorbance. This suggests that LiNO_3_ efficiently reduces the presence of disulfide radical anions and solvated electrons, as has previously been observed for sodium nitrate at 100 K (De la Mora *et al.*, 2011 ▶). For the crystalline HEWL system, a 12 s time series is shown in Fig. 6 ▶. Here both peaks are displayed and the initial rapid rise of the solvated electron absorption is followed by a slow decay which apparently feeds the disulfide bond reduction.

As further evidence of the scavenging power of nitrate, irradiation of an insulin crystal at 100 K resulted in a large absorption around 400 nm which was subsequently seen to be quenched in a crystal which had been soaked in 0.5 *M* LiNO_3_ [Figs. 7(*a*)–7(*d*) ▶]. The 580 nm peak showed an initial rise in the native sample, but, as with the HEWL crystal described above, decayed with time (Fig. 7*b* ▶).

### 3.3. Computational chemistry

In an attempt to identify the carriers of the various absorption features revealed by the microspectrophotometry investigations, characteristics of the radiation chemistry of glycerol were first probed. Further studies were performed on the likely transients from chloride and acetate-containing solutions.

The computations, using the procedure defined in §2.3 and thus denoted IEFPCM-B3LYP/6-311+G(d,p), revealed little (<8 kJ mol^–1^) difference in the energies of the radicals derived from H-abstraction by the radiation-generated hydroxyl radicals at the central or terminal carbon of glycerol. This 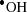 abstraction reaction is computed to be exothermic by about 100 kJ mol^–1^. On the other hand, H abstraction from the hydroxyl groups is less favourable, with a computed energy gain of about 70 kJ mol^–1^, so that oxygen-centred radicals are presumably less prevalent. Subsequent dehydration of the resulting C-centred radicals, computed to be an energetically favoured process at RT with a release in free energy of around 90 kJ mol^–1^, leads to the almost isoenergetic HC(=O)–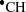
–CH_2_OH (denoted R1) and 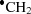
–C(=O)–CH_2_OH radicals.

None of the above-mentioned radicals show significant absorptions at wavelengths to the red of 200 nm (*i.e.* wavelengths longer than 200 nm). On the other hand, disproportionation of R1,
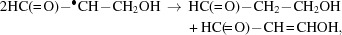
is computed to lead to 3-hydroxypropanal and the enol of malonic dialdehyde since the reaction is exothermic by 225 kJ mol^−1^. The latter compound is computed as above to have an intense absorption peak at 250 nm and indeed has been cited as the main contributor to absorbance in this region following radiolysis of polyol solutions in general (Ivanova *et al.*, 2009 ▶).

A similarly strong absorption band is predicted at 345 nm for the chloride dimer radical anion, 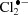, likely to be produced in the radiolysis of concentrated chloride solutions. This value is in agreement with literature reports (Jayson *et al.*, 1973 ▶). However, no such peak is detected here, suggesting either that its precursor may be otherwise scavenged or that the species may be too short-lived under the present conditions.

In addition to disproportionations, the carbon-centred radicals mentioned above can undergo dimerizations and cross reactions. Indeed, many of the products of sustained γ-radiolysis containing three, six and nine carbon atoms and varying numbers of O atoms have been previously identified by GCMS techniques (Baugh *et al.*, 1982 ▶). Also, in the presence of dissolved molecular oxygen, peroxide radicals are undoubtedly formed by addition at the radical sites. Computational investigations of typical structures of these products followed by evaluation of potential absorption peaks again showed little optical response at wavelengths longer than 200 nm.

## 4. Discussion

The debate concerning the effectiveness or otherwise of scavengers for use in MX continues, and possible reasons for the disparate results thus far reported have been investigated here. Sources of these conflicting conclusions from such studies include: choice of metric, radiation chemistry in the mother liquors and buffers utilized in MX, and uncertainties in the dose calculations, as well as inter-crystal variation (Owen *et al.*, 2012 ▶).

At present there is no unanimously agreed-upon global damage metric, which can make determining and comparing the effectiveness of different scavengers contentious. This is a significant challenge in the field since, as detailed above, different groups have produced conflicting results about the effectiveness of the same scavengers at the same temperatures in MX. The analyses presented here for sodium nitrate at 100 K show that the same data give different results and thus give rise to contrasting conclusions when analysed with different metrics. As has been observed by a number of researchers, there is also significant variation across crystals of the same protein which have nominally undergone the same treatment. It seems apparent that unless a clear enhancement of a factor of two or more in dose tolerance can be established, scavenger results should be treated with caution. Small positive effects are less likely to be reproducible and thus are not useful for general application in MX.

The fact that the global metrics *R*_d_, *B*_rel_ and *I*/*I*_0_ give different results is likely to be because these metrics are reporting on different properties of the crystal. Since *R*_d_ is calculated following data scaling, and gives the *R* values between reflections measured at different doses, this metric is dominated by non-isomorphism effects caused by unit-cell expansion, movement of the molecule within the unit cell, and structural changes due to specific damage. *B*_rel_ gives the change in overall scaling parameter for the wedge of data/dataset and is an indication of the average increase in disorder of the molecules in the crystal. Conversely, *I*/*I*_0_ is a direct measure of the diffraction strength of the crystal and is thus easier to relate directly to the quality and resolution of the electron density maps.

The analysis of the radiation chemistry occurring in the HEWL mother liquor was enabled by an innovative combination of technology; the humidity-controlling device permitted RT solutions to be tested using on-line microspectrophotometry without sample dehydration. The results demonstrate that irradiation of the HEWL cryoprotected mother liquor at RT causes an accumulation of species that absorb at 270 nm and 470 nm. These peaks were not observed when irradiation occurs at 100 K and this is probably a result of limited radiation chemistry during data collection due to reduced diffusion rates of the more bulky reactive radicals at these low temperatures. Since at RT the peaks only appear in the presence of glycerol, it is likely that glycerol radiolysis contributes in some way. At present the species that give rise to these peaks are unknown, but the results suggest that the peak at 470 nm is a consequence of both glycerol and NaCl radiation chemistry, whereas the peak at 270 nm is a consequence of both glycerol and NaAc radiation chemistry. Obvious candidates such as the dichloride radical anion (Cl_2_^−^) and the acetate radical (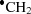
CO_2_^−^) have unrelated absorption maxima, with peaks at 340 nm and 350 nm, respectively. Peaks at these wavelengths were not observed in these experiments. Of course, the formation of both of these species is in competition with glycerol, present in much greater abundance, for the reactive hydroxyl radicals which are a primary product of X-ray irradiation.

Extensive computational exploration of the radiation chemistry of glycerol-containing solutions showed a thermochemically feasible route to the production of MDA which absorbs strongly around 250 nm.

The 470 nm peak observed following irradiation of the cryoprotected mother liquor at RT is quenched by the addition of 0.5 *M* sodium nitrate. The radiation chemistry of nitrate in dilute aqueous solution is complex, involving both radicals and radical ions (Gräzel *et al.*, 1970 ▶). One-electron reduction of nitrate by radiolytically generated hydrated electrons is very fast (*k* = 9.7 × 10^9^ *M* ^−1^ s^−1^) and the species formed, NO_3_^2−^, typically decomposes *via* an acid-catalysed equilibrium to give NO_2_ (Grätzel *et al.*, 1970 ▶). The resulting radical rapidly reacts with hydroxyl to give nitric acid, HNO_3_, which can presumably deprotonate to regenerate the nitrate ion. Similar reactions no doubt proceed in the mother liquor, though many other sinks, such as glycerol and chloride ion, are available for the hydroxyl radical. However, nitrate remains as an effective scavenger for electrons and can be predicted to quench specific damage otherwise caused by these agents. On the other hand the radicals formed upon reaction of nitrate with electrons can themselves, at least at RT, damage proteins, for example by addition to aromatic residues (Shi *et al.*, 2011 ▶). It is less clear then that any reduction in global damage might be expected, as indeed borne out by our in-house RT tests (see above). However, these products are presumably not mobile at 100 K where nitrate has been shown to be very effective in quenching specific damage to disulfide bonds in HEWL crystals and offered some reduction in the rate of global damage (De la Mora *et al.*, 2011 ▶). In fact, further support for the scavenging role of nitrate at 100 K is evident in the results presented by Macedo *et al.* (2009 ▶) who observed differences in the absorbance of cryobuffers intended for use in cryocooling azurin and myoglobin crystals though did not comment on their cause. No peak attributable to the solvated electron was observed in the azurin cryobuffer, likely due to the presence of 1 *M* lithium nitrate as a precipitant.

In order to rationalize the disparate scavenger studies summarized in Tables 1 ▶ and S1, it is thus of crucial importance to be aware that many crystallization buffers already contain efficient electron or 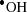 radical scavengers. 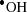 reaction rate constants in aqueous solutions at RT for the 14 salts and 14 aqueous organic compounds most frequently reported in the PDB (http://www.douglas.co.uk/top14.htm) are given in Table 4 ▶. On the other hand, none of these compounds react at significant rates with the hydrated electron. Clearly, careful account of the various concentrations of all cryobuffer and mother liquor components must be made in order to acquire consistent quantitative data on scavenger efficacy.

An example of the potential pitfalls in drawing conclusions from scavenger studies where crystallization agents with scavenging properties are included in the buffer is apparent from a study of the information collated in Table S1. As already mentioned, at 100 K, only electron and hole scavengers are expected to be effective. Macedo *et al.* (2009 ▶) reported that 0.1 *M* potassium hexacyanoferrate(III) (KF) was effective in quenching reduction of the copper in soaked azurin crystals, as shown by the persistence of a 632 nm absorbance peak characteristic of the non-reduced form for longer than it survived in native crystals. However, the crystallization buffer contained 0.5 *M* LiNO_3_, which may well have contributed to the observed effect by scavenging electrons which would otherwise have reduced the copper bound to the azurin. This would have been true for all the scavengers tested on azurin in that study, but perhaps the scavengers reported as ineffective (see Table S1) in the Macedo *et al.* study were in fact sensitizing the crystals and the LiNO_3_ was compensating for their effect. However, the authors also reported that KF decreased the rate of reduction of Fe^3+^ to Fe^2+^ in myoglobin crystals, which were not grown in LiNO_3_, so the picture is once again not completely clear.

Cryoprotectants such as glycerol also interact rapidly with hydroxyl radicals and would potentially cloud any experimental attempts to intercept the radical at low temperatures. In addition, under the usual crystallographic cryoconditions (100 K), 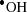 is thought to be immobile, and indeed a spectrum putatively assigned to the radical has just been reported in X-irradiated amorphous ice (Owen *et al.*, 2012 ▶). Thus hydroxyl radical scavengers would not be expected to give any extra protection at 100 K.

A corollary to the debate around scavenger efficacy is pertinent to the question of whether or not a dose limit related to the decay of diffraction intensity really exists for protein crystals. The points raised above imply that chemical modification of the experimental dose limit of 30 MGy determined at 100 K (Owen *et al.*, 2006 ▶) is indeed possible, but only if an electron or hole scavenger is present, since these are thought to be the only mobile species contributing to damage at this temperature.

It is clear that more studies are required to understand the radiation chemistry that is occurring at RT in these samples; however, the basic methodology has now been established for using on-line microspectrophotometry in conjunction with a humidifying device to look at radiation chemistry in RT MX. A more refined understanding of both this and the mechanisms dominant at 100 K will lead to sharper insights into the radiation damage dependent factors limiting data collection under cryoconditions.

## 5. Conclusions

The conflicting results from scavenger studies most probably arise from a number of factors, which include the use of different metrics, mother liquor radiation chemistry, and, of course, uncertainties in dose estimation.

Results from several studies certainly indicate the efficacy of scavengers in reducing certain types of specific damage both at cryotemperatures and RT, and of reducing the rate of global damage by more than a factor of two at RT. From the measurements reported above at RT using a humidity-controlling device and a microspectrophotometer, it is clear that the production of various species can be quenched by the addition of radical scavengers. However, it is less clear that this observation can be translated into a significant gain in crystal dose tolerance. Apart from 1,4-benzoquinone at RT, no scavenger at RT or 100 K has been reported to increase crystal dose tolerance by more than a factor of two. However, it is worth considering their use for specific cases.

Constituents of many crystallization buffers clearly interact with the various radiation-induced radicals which would otherwise increase the rate of specific damage. Conversely, screening the most common crystallization conditions for components that could intercept these reactive species may provide crystallographers with valuable information that may go some way toward decreasing radiation damage and enabling clear guidelines to be suggested for the scavenger approach to its mitigation.

Likely sources for some of the absorption profiles observed in microspectrophotometry have been identified using computational chemistry, but a clear identification of all of the peaks observed in the mother liquor components has remained elusive thus far.

Scavengers can only be useful as a mitigation strategy if clearer conclusions emerge and, with them, some general guidelines can be offered to MX experimenters on what might be attempted in order to extend the irradiation life (dose tolerance) of their crystals.

## Supplementary Material

Supplementary material file. DOI: 10.1107/S0909049512046237/xh5038sup1.pdf

## Figures and Tables

**Table 1:** Mother liquor conditions and results for reported MX scavenger studies where the results from different researchers are inconsistent Int = introduction: C = co-crystal, S = soak, N/A = not applicable. Damage: G = global damage, Sp = specific damage. Res = response: N = null, P = positive, S = sensitizing, U = unclear. Damage metrics: A### nm = absorbance peak detected by microspectrophotometry at the specified wavelength, |*F*_*n*_ − *F*_0_| = difference electron density maps calculated from the difference in structure factors for the *n*th dataset and first dataset. The other metrics are defined in the text.

Scavenger			Concentration of scavenger		Temperature		System†		Int		Conditions		Damage		Metric	Res
1,4-Benzoquinone			0.4 *M*		100 K		Disulfide/thiol model solutions^*a*^		N/A		0.1 *M* cystine, 0.75 *M* NaOH, 30% *v/v* EG, pH 13.3		Sp		A400 nm	P
			0.5 *M*		RT		Tetragonal HEWL crystals^*b*^		S		0.1 *M* NaAc pH 4.5, 4–8% *w/v* NaCl		G		Average *I*/*I*_0_	P
			Saturated		100 K		Azurin crystals^*c*^		S		5 m*M* NaAc pH 5.8, 1.85–1.95 *M* (NH_4_)_2_SO_4_, 0.5 *M* LiNO_3_		Sp		A632 nm	N
			Saturated		100 K		Myoglobin crystals^*c*^		S		50 m*M* Tris-HCl pH 7.2–7.4, 1.5–1.6 *M* (NH_4_)_2_SO_4_, 2.25% *v/v* PEG		Sp		A413–A427 nm, A500–A700 nm	N
2-Hydroxyethyl methacrylate (HEMA)			0.01 *M*		RT		Tetragonal HEWL crystals^*d*^		C		0.5 *M* NaCl		G		Δ*B*_rel_	S
			0.01 *M*		100 K		Tetragonal HEWL crystals^*d*^		C		0.5 *M* NaCl		G		Δ*B*_rel_	N
Ascorbate			>0.3 *M*		100 K		Tetragonal HEWL crystals^*e*^		C		0.2 *M* NaAc pH 4.7 3–7% *w/v* NaCl, 20% *v/v* glycerol		Sp		A400 nm	P
			0.5 *M*		100 K		N9 neuraminidase crystals^*f*^		S		1.7 *M* potassium phosphate 40% *v/v* glycerol		G		Average *I*/*I*_0_, unit cell	P
													Sp		|*F*_*n*_| − |*F*_0_|	
			0.8 *M*		92 K		Free SeMet-containing solutions^*g*^		N/A		25 m*M* SeMet, 25% *v/v* glycerol		Sp		XANES *D*_1/2_	P
			0.3 *M*		100 K		Disulfide/thiol model solutions^*a*^		N/A		0.1 *M* cystine, 0.75 *M* NaOH, 30% *v/v* EG, pH 12.9		Sp		A400 nm	P
			0.5 *M*		RT		Tetragonal HEWL crystals^*b*^		C		0.1 *M* NaAc pH 4.5, 4–8% *w/v* NaCl		G		Average *I*/*I*_0_	P
													Sp		|*F*_*n*_| − |*F*_0_|	P
			0.2 *M*		100 K		Azurin crystals^*c*^		S		5 m*M* NaAc pH 5.8, 1.85–1.95 *M* (NH_4_)_2_SO_4_, 0.5 *M* LiNO_3_		Sp		A632 nm	P
													Sp		|*F*_*n*_| − |*F*_0_|	P
													G		Average *I*/*I*_0_	P
			0.2 *M*		100 K		Myoglobin crystals^*c*^		S		50 m*M* Tris-HCl pH 7.2–7.4, 1.5–1.6 *M* (NH_4_)_2_SO_4_, 2.25% *v/v* PEG		Sp		A413–A427 nm, A500–A700 nm	N
			1.0 *M*		100 K		Tetragonal HEWL crystals^*h*^		C		0.1 *M* NaAc pH 4.7, 10% *w/v* NaCl, 30% *v/v* glycerol		Sp		|*F*_*n*_| − |*F*_0_|	P
													G		Average *I*/*I*_0_	P
			0.1 *M*		RT, 100 K		Tetragonal HEWL crystals^*d*^		C		0.5 *M* NaCl		G		Δ*B*_rel_	N
Cysteine			0.2 *M*		100 K		Azurin crystals^*c*^		S		5 m*M* NaAc pH 5.8, 1.85–1.95 *M* (NH_4_)_2_SO_4_, 0.5 *M* LiNO_3_		Sp		A632 nm	N
															|*F*_*n*_| − |*F*_0_|	N
			0.2 *M*		100 K		Myoglobin crystals^*c*^		S		50 m*M* Tris-HCl pH 7.2–7.4, 1.5–1.6 *M* (NH_4_)_2_SO_4_, 2.25% *v/v* PEG		Sp		A413–A427 nm, A500–A700 nm	N
			0.1 *M*		RT		Tetragonal HEWL crystals^*d*^		C		0.5 *M* NaCl		G		Δ*B*_rel_	S
			0.1 *M*		100 K		Tetragonal HEWL crystals^*d*^		C		0.5 *M* NaCl		G		Δ*B*_rel_	N
DTNB			0.2 *M*		100 K		Tetragonal HEWL crystals^*i*^		S		25 m*M* NaAc pH 4.5, 5% *w/v* NaCl		Sp		|*F*_*n*_| − |*F*_0_|	U
													G		*R*_d_	P
			0.2 *M*		100 K		PPE crystals^*i*^		S		50 m*M* NaAc pH 5.1, 100 m*M* sodium citrate, 20 m*M* CaCl_2_		Sp		|*F*_*n*_| − |*F*_0_|	P
													G		*R*_d_	P
			0.2 *M*		100 K		Thaumatin crystals^*i*^		S		50 m*M* ADA pH 6.5, 500 m*M* sodium/potassium tartrate		Sp		|*F*_*n*_| − |*F*_0_|	P
													G		*R*_d_	P
Glutathione (oxidized)			0.2 *M*		100 K		Tetragonal HEWL crystals^*i*^		S		25 m*M* NaAc pH 4.5, 5% *w/v* NaCl		G		*R*_d_	N
			0.2 *M*		100 K		PPE crystals^*i*^		S		50 m*M* NaAc pH 5.1, 100 m*M* sodium citrate, 20 m*M* CaCl_2_		Sp		|*F*_*n*_| − |*F*_0_|	N
													G		*R*_d_	S
			0.2 *M*		100 K		Thaumatin crystals^*i*^		S		50 m*M* ADA pH 6.5, 500 m*M* sodium/potassium tartrate		Sp		|*F*_*n*_| − |*F*_0_|	P
													G		*R*_d_	
HEPES			0.5 *M*		100 K		Disulfide/thiol model solutions^*a*^		N/A		0.1 *M* cystine, 0.5 *M* NaOH, 30% *v/v* EG, pH 9.66		Sp		A400 nm	N
			0.2 *M*		100 K		Azurin crystals^*c*^		S		5 m*M* NaAc pH 5.8, 1.85–1.95 *M* (NH_4_)_2_SO_4_, 0.5 *M* LiNO_3_		Sp		A632 nm	U
															|*F*_*n*_| − |*F*_0_|	U
Hydroquinone			0.1 *M*		RT		Tetragonal HEWL crystals^*d*^		C		0.5 *M* NaCl		G		Δ*B*_rel_	S
			0.1 *M*		100 K		Tetragonal HEWL crystals^*d*^		C		0.5 *M* NaCl		G		Δ*B*_rel_	N
Nicotinic acid			0.2 *M*		100 K		Tetragonal HEWL crystals^*i*^		S		25 m*M* NaAc pH 4.5, 5% *w/v* NaCl		Sp		|*F*_*n*_| − |*F*_0_|	U
													G		*R*_d_	P
			0.2 *M*		100 K		PPE crystals^*i*^		S		50 m*M* NaAc pH 5.1, 100 m*M* sodium citrate, 20 m*M* CaCl_2_		Sp		|*F*_*n*_| − |*F*_0_|	U
													G		*R*_d_	P
			0.2 *M*		100 K		Thaumatin crystals^*i*^		S		50 m*M* ADA pH 6.5, 500 m*M* sodium/potassium tartrate		Sp		|*F*_*n*_| − |*F*_0_|	P
													G		*R*_d_	P
			0.15 *M*		100 K		Bovine pancreatic trypsin crystals^*j*^		S		2.5 mg ml^−1^ benzamidine, 15 m*M* HEPES, pH 7.0 1.5 m*M* CaCl_2,_ 10% PEG 8 K 50 m*M* cacodylate pH 6.5 100 m*M* ammonium sulfate 7.5% glycerol		G		*R*_d_	N
*N*-*tert*-Butyl-α-phenylnitrone (PBN)			0.16 *M*		RT		Tetragonal HEWL crystals^*d*^		C		0.5 *M* NaCl		G		Δ*B*_rel_	S
			0.16 *M*		100 K		Tetragonal HEWL crystals^*d*^		C		0.5 *M* NaCl		G		Δ*B*_rel_	N
PEG 4000			15%		100 K		Canavalin crystals^*k*^		S		0.7% NaCl		G		Average *I*/*I*_0_	P
			20%		100 K		Fructose 1,6 diphos­phatase^*k*^		S		–		G		Average *I*/*I*_0_	P
			12%, 45%		100 K		Disulfide/thiol model solutions^*a*^		N/A		0.1 *M* cystine, 0.75 *M* NaOH, 30% *v/v* glycerol, pH 13.59, 13.70		Sp		A400 nm	N
			0.1 *M*		RT		Tetragonal HEWL crystals^*d*^		C		0.5 *M* NaCl		G		Δ*B*_rel_	S
			0.1 *M*		100 K		Tetragonal HEWL crystals^*d*^		C		0.5 *M* NaCl		G		Δ*B*_rel_	N
Reduced DTT			0.5 *M*		100 K		Disulfide/thiol model solutions^*a*^		N/A		0.1 *M* cystine, 0.5 *M* NaOH, 30% *v/v* EG, pH 9.5		Sp		A400 nm	U
Sodium nitrate			0.02 *M*		195 K		*β*-Galactosidase solutions^*l*^		N/A		0.01 *M* phosphate, pH 8.0		Sp		Mass of native polypeptide	U
			0.02 *M*		RT		β-Galactosidase solutions^*l*^		N/A		0.01 *M* phosphate, pH 8.0		Sp		Mass of native polypeptide	N
			1 *M*		92 K		Free SeMet-containing solutions^*g*^		N/A		25 m*M* SeMet, 25% *v/v* glycerol		Sp		XANES *D*_1/2_	P
			1%		40 K		Tetragonal HEWL crystals^*m*^		C		50 m*M* NaAc pH 4.5, 0.25 *M* NaCl 30% *v/v* EG		Sp		|*F*_*n*_| − |*F*_0_|	P
			0.5 *M*		100 K		Tetragonal HEWL crystals^*h*^		S		0.1 *M* NaAc pH 4.7, 10% *w/v* NaCl, 30% *v/v* glycerol		Sp		A400 nm	P
													Sp		|*F*_*n*_| − |*F*_0_|	P
													G		*I*/*I*_0_	P
			0.1 *M*		RT		Tetragonal HEWL crystals^*d*^		S		0.5 *M* NaCl		G		Δ*B*_rel_	P
			0.1 *M*		100 K		Tetragonal HEWL crystals^*d*^		S		0.5 *M* NaCl		G		Δ*B*_rel_	N
Styrene			0.002 *M*		RT		DOB immunoglobulin crystals^*n*,*o*^		C		70% 0.1 *M* sodium borate, pH 8.4		G		*I*/*I*_0_ for 2 or 3 reflections	P
			Saturated		100 K		Tetragonal HEWL crystals^*e*^		C		25 m*M* NaAc pH 4.5, 0.5 *M* NaCl, 30% MPEG 5K 10% *v/v* glycerol		Sp		|*F*_*n*_| − |*F*_0_|	N
													G		Average *I*/*I*_0_	N
			0.1 *M*		RT		Tetragonal HEWL crystals^*d*^		C		0.5 *M* NaCl		G		Δ*B*_rel_	S
			0.1 *M*		100 K		Tetragonal HEWL crystals^*d*^		C		0.5 *M* NaCl		G		Δ*B*_rel_	N
Thiourea			0.5 *M*		100 K		Disulfide/thiol model solutions^*a*^		N/A		0.1 *M* cystine, 0.25 *M* NaOH, 30% *v/v* EG, pH 10.87		Sp		A400 nm	U
			0.2 *M*		100 K		Azurin crystals^*c*^		S		5 m*M* NaAc pH 5.8, 1.85–1.95 *M* (NH_4_)_2_SO_4_, 0.5 *M* LiNO_3_		G		A632 nm	U
			0.4 *M*		RT, 100 K		Tetragonal HEWL crystals^*d*^		C		0.5 *M* NaCl		G		Δ*B*_rel_	N
Trehalose			1 *M*		100K		Disulfide/thiol model solutions^*a*^		N/A		0.1 *M* cystine, 0.25 *M* NaOH, 30% *v/v* glycerol, pH 12.1		Sp		A400 nm	N
Tris			0.02 *M*		RT		β-Galactosidase solutions^*l*^		N/A		0.01 *M* phosphate, pH 8.0		Sp		Mass of native polypeptide	P
			0.02 *M*		195 K		β-Galactosidase solutions^*l*^		N/A		0.01 *M* phosphate, pH 8.0		Sp		Mass of native polypeptide	N
			0.02 *M*		RT		β-Galactosidase lyophilisized powders^*l*^		N/A		0.01 *M* phosphate, pH 8.0		Sp		Mass of native polypeptide	P
			0.02 *M*		195 K		β-Galactosidase lyophilisized powders^*l*^		N/A		0.01 *M* phosphate, pH 8.0		Sp		Mass of native polypeptide	N

† ^*a*^Southworth-Davies & Garman (2007 ▶). ^*b*^Barker *et al.* (2009 ▶). ^*c*^Macedo *et al.* (2009 ▶). ^*d*^Kmetko *et al.* (2011 ▶). ^*e*^Murray & Garman (2002 ▶). ^*f*^Betts (2003 ▶). ^*g*^Holton (2007 ▶). ^*h*^De la Mora *et al.* (2011 ▶). ^*i*^Kauffmann *et al.* (2006 ▶). ^*j*^Nowak *et al.* (2009 ▶). ^*k*^Cascio *et al.* (1984 ▶). ^*l*^Audette-Stuart *et al.* (2005 ▶). ^*m*^Borek *et al.* (2007 ▶). ^*n*^Zaloga & Sarma (1974 ▶). ^*o*^Sarma & Zaloga (1975 ▶).

**Table 2:** Crystals from which data were previously collected at 100 K (De la Mora *et al.*, 2011 ▶) For data collection statistics see Table 2 of De la Mora *et al.* (2011 ▶).

Sample name	Crystal	Soaked/co-crystallized with scavenger	Scavenger (number of datasets)
Native HEWL	HEWL	N/A	None (6)
HEWL–ascorbate co-crystal	HEWL	Co-crystallized	Ascorbate (6)
HEWL + Nitrate I	HEWL	Soaked for 4 min	Sodium nitrate (final concentration 0.5 *M*) (5)
HEWL + Nitrate II	HEWL	Soaked for 4 min	Sodium nitrate (final concentration 0.5 *M*) (6)
HEWL + Nitrate8	HEWL	Soaked for 8 min	Sodium nitrate (final concentration 0.5 *M*) (6)

**(a):** The values of *D*_1/2_ and Δ*B*_rel_/Δ*D* (slope of *D versus B*_rel_ graph) resulting from the reanalysis described in the text.

Metric	Δ*B*_rel_ /Δ*D* (Å^2^ MGy^−1^)		*D*_1/2_ (MGy)
Data used for metric	*B*_rel_5	*B*_rel_DS	*I*/*I*_0_
Native HEWL	1.04	0.98	12.6
HEWL + Nitrate I	0.68	0.83	18.8
HEWL + Nitrate II	0.41	0.46	21.2
Mean of HEWL + Nitrate I and II	0.55	0.65	20.0
HEWL + Nitrate8	0.52	0.55	10.4
HEWL-ascorbate co-crystal	0.39	0.53	24.0

**(b):** The enhancement factors, *E*_*B*_ and *E*_*I*_, obtained using the two different metrics.†

Enhancement factor (a.u.)	*E*_*B*_	*E*_*B*_	*E*_*I*_
Metric used in reanalysis	*B*_rel_5	*B*_rel_DS	*I*/*I*_0_
HEWL + Nitrate I	1.53	1.18	1.44
HEWL + Nitrate II	2.54	2.13	1.68
Mean Nitrate I and II	1.90	1.52	1.59
HEWL + Nitrate8	2.01	1.77	0.83
HEWL–ascorbate co-crystal	2.65	1.86	1.90

† For *I*/*I*_0_, this is defined as the ratio of *D*_1/2_ (dose to half intensity) with scavenger added to *D*_1/2_ of the native. For the *B*_rel_ analysis, the enhancement factor is the ratio of Δ*B*_rel_/*ΔD* without scavenger to Δ*B*_rel_/Δ*D* with scavenger (thus a value >1 implies some protection).

**Table:** 

	No. of entries	Average concentration (% *v*/*v*)	Rate (*M* ^−1^ s^−1^)†
Organic precipitants (2503 samples)			
PEG 4K	710	21.1	∼3 × 10^9^
PEG 8K	488	18.1	"
PEG 3.5K	296	20.5	"
PEG 6K	212	16.8	"
MPD	193	38.6	∼6 × 10^8^
PEG 400	142	25.7	∼3 × 10^9^
PEG-MME 2000	65	22.7	"
PEG-MME 5000	63	20.0	"
PEG 1000	57	19.8	"
2-Propanol	48	18.0	2.0 × 10^9^
PEG 2000	45	22.3	∼3 × 10^9^
Ethylene glycol	43	20.5	2.4 × 10^9^
Ethanol	43	28.8	2.0 × 10^9^
PEG 10K	32	22.0	∼3 × 10^9^

**Table:** 

	No. of entries	Average concentration (*M*)	Rate (*M* ^−1^ s^−1^)†
Salt precipitant (1436 samples)			
Ammonium sulfate	900	1.9	*
Sodium chloride	124	1.7	3.0 × 10^9^
Sodium citrate	76	1.1	1.5 × 10^8^
Sodium/potassium phosphate	66	1.8	*
Lithium sulfate	63	1.4	*
Sodium formate	59	3.4	3.2 × 10^9^
Magnesium sulfate	29	1.7	*
Ammonium phosphate	29	1.5	*
Potassium phosphate	25	2.0	*
Sodium acetate	21	1.2	1.0 × 10^8^
Sodium/potassium tartrate	13	1.0	1.4 × 10^9^
Caesium chloride	11	2.7	3.0 × 10^9^
Potassium chloride	10	1.4	3.0 × 10^9^
Sodium phosphate	10	1.4	*

† Experimentally determined rates in dilute aqueous solution at RT (Buxton *et al.*, 1988 ▶). *Reaction too slow to be detected.

## Footnotes

1 Supplementary data for this paper are available from the IUCr electronic archives (Reference: XH5038). Services for accessing these data are described at the back of the journal.

2 All computations were performed with the *Gaussian09* software package (Frisch *et al.*, 2009; for reference see supplementary material).
