# Supplementary material for: To scavenge or not to scavenge, that is STILL the question
Source: J Synchrotron Radiat. 2012 Dec 5;20(Pt 1):23–36. doi: 10.1107/S0909049512046237 (PMC3526919; doi:10.1107/S0909049512046237)
Supplement: Supplementary file 1 [file s-20-00023-sup1.pdf]

Supplementary Material for xh5038. Allan et al. JSR (2013) 20.

To scavenge or not to scavenge, that is STILL the question.

Elizabeth G. Allan, Melissa C. Kander, Ian Carmichael and Elspeth F. Garman

Table S1 Mother liquor conditions and results for MX scavenger studies reported in the literature to date.

[a] Int = introduction; C = co-crystal, S = soak, N/A = not applicable; [b] G = global damage, Sp = specific damage.
[c] Metrics: A### nm = absorbance peak detected by microspectrophotometry at the specified wavelength, $|F_n-F_0|$= difference electron density maps calculated from the difference in structure factors for the nth dataset and first dataset. The other metrics are defined in the main paper.
[x] Res = response; N = null, P = positive, S = sensitizing, U = unclear.

Scavenger	Concentration of scavenger	Temperature	System	Int[a]	Conditions	Damage[b]	Metric[c]	Res[x]
**1,4-benzoquinone**	0.4 M	100 K	Disulphide/thiol model solutions[r]	N/A	0.1 M cystine,<br>0.75 M NaOH,<br>30% *v/v* EG, pH 13.3	Sp	A400 nm	P
	0.5 M	RT	Tetragonal HEWL crystals[e]	S	0.1 M NaAc pH 4.5,<br>4-8% *w/v* NaCl	G	Average I/I_0	P
	Saturated	100 K	Azurin crystals[n]	S	5 mM NaAc pH 5.8,<br>1.85 M - 1.95 M $(NH4)_2SO_4$,<br>0.5 M $LiNO_3$,	Sp	A632 nm	N
	Saturated	100 K	Myoglobin crystals[n]	S	50 mM Tris-HCl pH 7.2-7.4,<br>1.5 M - 1.6 M $(NH4)_2SO_4$, 2.25% *v/v* PEG	Sp	A413 – A427 nm,<br>A500 – A700 nm	N
**1,4-dithiothreitol**	0.1 M	RT, 100K	Tetragonal HEWL crystals[m]	C	0.5 M NaCl	G	ΔB_{rel}	N
**2,3-dichloro-1,4-naphtoquinone**	Saturated	100 K	Azurin crystals[n]	S	5 mM NaAc pH 5.8,<br>1.85 M - 1.95 M $(NH4)_2SO_4$,	Sp	A632 nm	U

					0.5 M $LiNO_3$		$\|F_n\| - \|F_0\|$	U
**2,3-dichloro-5,6-dicyano-1,4-benzoquinone**	Saturated	100 K	Azurin crystals[n]	S	5 mM NaAc pH 5.8, 1.85 M - 1.95 M $(NH4)_2SO_4$, 0.5 M $LiNO_3$	Sp	A632 nm	N
	Saturated	100 K	Myoglobin crystals[n]	S	50 mM Tris-HCl pH 7.2-7.4, 1.5 M - 1.6 M $(NH4)_2SO_4$, 2.25% *v/v* PEG	Sp	A413 – A427 nm, A500 – A700 nm	N
**2,6-dichloroindophenol**	Saturated	100 K	Azurin crystals[n]	S	5 mM NaAc pH 5.8, 1.85 M - 1.95 M $(NH4)_2SO_4$, 0.5 M $LiNO_3$	Sp	A632 nm	N
	Saturated	100 K	Myoglobin crystals[n]	S	50 mM Tris-HCl pH 7.2-7.4, 1.5 M - 1.6 M $(NH4)_2SO_4$, 2.25% *v/v* PEG	Sp	A413 – A427 nm, A500 – A700 nm	N
**2-hydroxyethyl methacrylate (HEMA)**	0.01 M	RT	Tetragonal HEWL crystals[m]	C	0.5 M NaCl	G	ΔB_{rel}	S
	0.01 M	100 K	Tetragonal HEWL crystals[m]	C	0.5 M NaCl	G	ΔB_{rel}	N
**2-nitroimidazole**	Saturated	100 K	Azurin crystals[n]	S	5 mM NaAc pH 5.8, 1.85 M - 1.95 M $(NH4)_2SO_4$, 0.5 M $LiNO_3$	Sp<br>G	$\|F_n\| - \|F_0\|$<br>Average I/I_0	N<br>N
**3,4,5,6-tetrachloro-1,2-benzoquinone**	Saturated	100 K	Azurin crystals[n]	S	5 mM NaAc pH 5.8, 1.85 M - 1.95 M $(NH4)_2SO_4$, 0.5 M $LiNO_3$	S	A632 nm	N
	Saturated	100 K	Myoglobin crystals[n]	S	50 mM Tris-HCl pH 7.2-7.4, 1.5 M - 1.6 M $(NH4)_2SO_4$, 2.25% *v/v* PEG	S	A413 – A427 nm, A500 – A700 nm	N
**4-methoxyphenol (MEHQ)**	1.6 M	RT, 100 K	Tetragonal HEWL crystals[m]	C	0.5 M NaCl	G	ΔB_{rel}	N

**5,5,-dithio-bis-(2-nitrobenzoic acid)**	Saturated	100 K	Azurin crystals[n]	S	5 mM NaAc pH 5.8, 1.85 M - 1.95 M $(NH4)_2SO_4$, 0.5 M $LiNO_3$	Sp	A632 nm	N
	Saturated	100 K	Myoglobin crystals[n]	S	50 mM Tris-HCl pH 7.2-7.4, 1.5 M - 1.6 M $(NH4)_2SO_4$, 2.25% *v/v* PEG	Sp	A413 – A427 nm, A500 – A700 nm	N
**Acetone**	0.5 M	100K	Disulphide/thiol model solutions[r]	N/A	0.1 M cystine 0.5 M NaOH, 30% *v/v* EG, pH 13.2	Sp	A400 nm	N
**Anthraquinone**	Saturated	100 K	Azurin crystals[n]	S	5 mM NaAc pH 5.8, 1.85 M - 1.95 M $(NH4)_2SO_4$, 0.5 M $LiNO_3$	Sp	A632 nm	N
	Saturated	100K	Myoglobin crystals[n]	S	50 M Tris-HCl pH 7.2-7.4, 1.5 M - 1.6 M $(NH4)_2SO_4$, 2.25% *v/v* PEG	Sp	A413 – A427 nm, A500 – A700 nm	N
**Ascorbate**	> 0.3 M	100 K	Tetragonal HEWL crystals[o]	C	0.2 M NaAc pH 4.7 3-7 % *w/*v NaCl, 20 % *v/v* glycerol	Sp	A400 nm	P
	0.5 M	100 K	N9 neuraminidase crystals[f]	S	1.7 M potassium phosphate 40% *v/v* glycerol	G, Sp	Average I/I_0, unit cell $\|F_n\| - \|F_0\|$	P
	0.8 M	92 K	Free SeMet-containing solutions[j]	N/A	25 mM SeMet, 25% *v/v* glycerol,	Sp	XANES $D_{1/2}$	P
	0.3 M	100 K	Disulphide/thiol model solutions[r]	N/A	0.1 M cystine, 0.75M NaOH, 30% *v/v* EG, pH 12.9	Sp	A400 nm	P

	0.5 M	RT	Tetragonal HEWL crystals[e]	C	0.1 M NaAc pH 4.5, 4-8% *w/v* NaCl	G	Average I/I_0	P
						Sp	$\|F_n\| - \|F_0\|$	P
	0.2 M	100 K	Azurin crystals[n]	S	5 mM NaAc pH 5.8, 1.85 M - 1.95 M $(NH4)_2SO_4$, 0.5 M $LiNO_3$	Sp	A632 nm	P
						Sp	$\|F_n\| - \|F_0\|$	P
						G	Average I/I_0	P
	0.2 M	100 K	Myoglobin crystals[n]	S	50 mM Tris-HCl pH 7.2-7.4, 1.5 M - 1.6 M $(NH4)_2SO_4$, 2.25% *v/v* PEG	Sp	A413 – A427 nm, A500 – A700 nm	N
	1.0 M	100 K	Tetragonal HEWL crystals[i]	C	0.1 M NaAc pH 4.7, 10% *w/v* NaCl, 30% *v/v* glycerol	Sp	$\|F_n\| - \|F_0\|$	P
						G	Average I/I_0	P
	0.1 M	RT, 100 K	Tetragonal HEWL crystals[m]	C	0.5 M NaCl	G	ΔB_{rel}	N
**Butylated hydroxytoluene**	0.5 M	100 K	Disulphide/thiol model solutions[r]	N/A	0.1 M cystine, 0.5 M NaOH, 30% *v/v* EG, pH 13.1	S	A400 nm	N
**Cystamine**	0.2 M	RT, 100K	Tetragonal HEWL crystals[m]	S	0.5 M NaCl	G	ΔB_{rel}	N
**Cysteamine**	0.1 M	RT, 100K	Tetragonal HEWL crystals[m]	S	0.5 M NaCl	G	ΔB_{rel}	N
**Cysteine**	0.2 M	100 K	Azurin crystals[n]	S	5 mM NaAc pH 5.8, 1.85 M - 1.95 M $(NH4)_2SO_4$, 0.5 M $LiNO_3$	Sp	A632 nm	N
							$\|F_n\| - \|F_0\|$	N
	0.2 M	100 K	Myoglobin crystals[n]	S	50 mM Tris-HCl pH 7.2-7.4, 1.5 M - 1.6 M $(NH4)_2SO_4$, 2.25% *v/v* PEG	Sp	A413 – A427 nm, A500 – A700 nm	N

	0.1 M	RT	Tetragonal HEWL crystals[m]	C	0.5 M NaCl	G	ΔB_{rel}	S
	0.1 M	100 K	Tetragonal HEWL crystals[m]	C	0.5 M NaCl	G	ΔB_{rel}	N
	100 %	RT, 100 K	Tetragonal HEWL crystals[m]	C	0.5 M NaCl	G	ΔB_{rel}	N
**DTNB**	0.2 M	100 K	Tetragonal HEWL crystals[l]	S	25 mM NaAc pH 4.5,<br>5% *w/v* NaCl	Sp<br>G	$\|F_n\| - \|F_0\|$<br>R_d	U<br>P
	0.2 M	100 K	PPE crystals[l]	S	50 mM NaAc pH 5.1,<br>100 mM sodium citrate,<br>20 mM $CaCl_2$	Sp<br>G	$\|F_n\| - \|F_0\|$<br>R_d	P<br>P
	0.2 M	100 K	Thaumatin crystals[l]	S	50 mM ADA pH 6.5,<br>500 mM sodium/potassium tartrate	Sp<br>G	$\|Fn_n\| - \|F_0\|$<br>R_d	P<br>P
**Ethanol**	0.5 M	100 K	Disulphide/thiol model solutions[r]	N/A	0.1 M cystine,<br>0.5 M NaOH,<br>30% *v/v* EG, pH 12.96	S	A400 nm	N
**$Fe(CN)_6^{-3}$**	Not specified	77 K	Deoxyhaemoglobin[k]	N/A	Not specified	Sp	EPR	P
**Fluorescein**	Saturated	100 K	Azurin crystals[n]	S	5 mM NaAc pH 5.8,<br>1.85 M - 1.95 M $(NH4)_2SO_4$,<br>0.5 M $LiNO_3$	Sp	A632 nm	N
	Saturated	100 K	Myoglobin crystals[n]	S	50 mM Tris-HCl pH 7.2-7.4,<br>1.5 M - 1.6 M $(NH4)_2SO_4$,<br>2.25% *v/v* PEG	Sp	A413 – A427 nm,<br>A500 – A700 nm	N
**Glucose**	1 M	100 K	Disulphide/thiol model solutions[r]	N/A	0.1 M cystine,<br>0.25 M NaOH,<br>30% *v/v* glycerol, pH 12.5	Sp	A400 nm	N

	0.5 M	100 K	N9 Neuraminidase crystals[f]	S	1.7 M potassium phosphate<br>40% *v/v* glycerol	G<br>Sp	Average I/I_0, unit cell<br>$\|F_n\| - \|F_0\|$	S
**Glutathione (oxidised)**	0.2 M	100 K	Tetragonal HEWL crystals[l]	S	25 mM NaAc pH 4.5,<br>5% *w/v* NaCl	G	R_d	N
	0.2 M	100 K	PPE crystals[l]	S	50 mM NaAc pH 5.1,<br>100 mM sodium citrate,<br>20 mM $CaCl_2$	Sp<br>G	$\|F_n\| - \|F_0\|$<br>R_d	N<br>S
	0.2 M	100 K	Thaumatin crystals[l]	S	50 mM ADA pH 6.5,<br>500 mM sodium/potassium tartrate	Sp<br>G	$\|F_n\| - \|F_0\|$<br>R_d	P
**Glutathione (reduced)**	0.1 M	RT, 100K	Tetragonal HEWL crystals[m]	C	0.5 M NaCl	G	ΔB_{rel}	N
**HEPES**	0.5 M	100 K	Disulphide/thiol model solutions[r]	N/A	0.1 M cystine,<br>0.5 M NaOH,<br>30% *v/v* EG, pH 9.66	Sp	A400 nm	N
	0.2 M	100 K	Azurin crystals[n]	S	5 mM NaAc pH 5.8,<br>1.85 M - 1.95 M $(NH4)_2SO_4$,<br>0.5 M $LiNO_3$	Sp	A632 nm<br>$\|F_n\| - \|F_0\|$	U<br>U
**Hydroquinone**	0.1 M	RT	Tetragonal HEWL crystals[m]	C	0.5 M NaCl	G	ΔB_{rel}	S
	0.1 M	100 K	Tetragonal HEWL crystals[m]	C	0.5 M NaCl	G	ΔB_{rel}	N

**Malic acid anhydride**	0.1 M	100 K	Azurin crystals[n]	S	5 mM NaAc pH 5.8,<br>1.85 M - 1.95 M $(NH4)_2SO_4$,<br>0.5 M $LiNO_3$	Sp	A632 nm	N
	0.2 M	100 K	Myoglobin crystals[n]	S	50 mM Tris-HCl pH 7.2-7.4,<br>1.5 M - 1.6 M $(NH4)_2SO_4$,<br>2.25% *v/v* PEG	Sp	A413 – A427 nm,<br>A500 – A700 nm	N
**Maltose**	1 M	100 K	Disulphide/thiol model solutions[r]	N/A	0.1 M cystine,<br>0.25 M NaOH,<br>30% *v/v* glycerol, pH 8.01	Sp	A400 nm	N
**Maltotriose**	1 M	100 K	Disulphide/thiol model solutions[r]	N/A	0.1 M cystine,<br>0.25 M NaOH,<br>30% *v/v* glycerol, pH 8.17	Sp	A400 nm	N
**Methimazole**	0.5 M	RT, 100 K	Tetragonal HEWL crystals[m]	C	0.5 M NaCl	G	ΔB_{rel}	N
**Methylacrylate**	0.5 M	100 K	Disulphide/thiol model solutions[r]	N/A	0.1 M cystine,<br>0.5 M NaOH,<br>30% *v/v* EG, pH 7.69	G	A400nm	N
**Nicotinic acid**	0.2 M	100 K	Tetragonal HEWL crystals[l]	S	25 mM NaAc pH 4.5,<br>5% *w/v* NaCl	Sp<br>G	$\|F_n\| - \|F_0\|$<br>R_d	U<br>P
	0.2 M	100 K	PPE crystals[l]	S	50 mM NaAc pH 5.1,<br>100 mM sodium citrate,<br>20 mM $CaCl_2$	Sp<br>G	$\|F_n\| - \|F_0\|$<br>R_d	U<br>P
	0.2 M	100 K	Thaumatin crystals[l]	S	50 mM ADA pH 6.5,<br>500 mM sodium/potassium tartrate	Sp<br>G	$\|F_n\| - \|F_0\|$<br>R_d	P<br>P

	0.15 M	100 K	Bovine pancreatic trypsin crystals[p]	S	2.5 mg/ml benzamidine,<br>15 mM HEPES, pH 7.0<br>1.5 mM $CaCl_2$<br>10% PEG 8K<br>50 mM cacodylate pH 6.5<br>100 mM ammonium sulphate<br>7.5% glycerol	G	R_d	N
**Nicotinamide adenine dinucleotide (NADH)**	0.05 M	RT, 100 K	Tetragonal HEWL crystals[m]	C	0.5 M NaCl	G	ΔB_{rel}	N
**N-*tert*-Butyl-α-phenylnitrone (PBN)**	0.16 M	RT	Tetragonal HEWL crystals[m]	C	0.5 M NaCl	G	ΔB_{rel}	S
	0.16 M	100 K	Tetragonal HEWL crystals[m]	C	0.5 M NaCl	G	ΔB_{rel}	N
**PEG 4000**	15%	RT	Canavalin crystals[h]	S	0.7 % NaCl	G	Average I/I_0	P
	20%	RT	Fructose 1,6 diphosphatase[h]	S	-	G	Average I/I_0	P
	12%, 45%	100 K	Disulphide/thiol model solutions[r]	N/A	0.1 M cystine,<br>0.75 M NaOH,<br>30% *v/v* glycerol, pH 13.59, 13.70	Sp	A400 nm	N
	0.1 M	RT	Tetragonal HEWL crystals[m]	C	0.5 M NaCl	G	ΔB_{rel}	S
	0.1 M	100 K	Tetragonal HEWL crystals[m]	C	0.5 M NaCl	G	ΔB_{rel}	N
**Potassium hexacyanoferrate(III)**	0.1 M	100 K	Azurin crystals[n]	S	5 mM NaAc pH 5.8,<br>1.85 M - 1.95 M $(NH4)_2SO_4$,<br>0.5 M $LiNO_3$	Sp	A632 nm	P

	1.5 M	100 K	Myoglobin crystals[n]	S	50 mM Tris-HCl pH 7.2-7.4, 1.5 M - 1.6 M $(NH4)_2SO_4$, 2.25% *v/v* PEG	Sp	A413 – A427 nm, A500 – A700 nm	P
**Reduced DTT**	0.5 M	100 K	Disulphide/thiol model solutions[r]	N/A	0.1 M cystine, 0.5 M NaOH, 30% *v/v* EG, pH 9.5	Sp	A400 nm	U
**Sodium bromide**	0.4 M	RT, 100 K	Tetragonal HEWL crystals[m]	S	0.5 M NaCl	G	ΔB_{rel}	N
**Sodium iodide**	1 M	RT, 100 K	Tetragonal HEWL crystals[m]	S	0.5 M NaCl	G	ΔB_{rel}	N
**Sodium nitrate**	0.02 M	195 K	*β*-galactosidase solutions[d]	N/A	0.01 M phosphate, pH 8.0	Sp	Mass of native polypeptide	U
	0.02 M	RT	*β*-galactosidase solutions[d]	N/A	0.01 M phosphate, pH 8.0	Sp	Mass of native polypeptide	N
	1 M	92 K	Free SeMet-containing solutions[j]	N/A	25 mM SeMet, 25% *v/v* glycerol	Sp	XANES $D_{1/2}$	P
	1%	40 K	Tetragonal HEWL crystals[g]	C	50 mM NaAc pH 4.5, 0.25 M NaCl 30% *v/v* EG	Sp	$\|F_n\| - \|F_0\|$	P
	0.5 M	100 K	Tetragonal HEWL crystals[i]	S	0.1M NaAc pH 4.7, 10% *w/v* NaCl, 30% *v/v* glycerol	Sp	A400 nm	P
						Sp	$\|F_n\| - \|F_0\|$	P
						G	I/I_0	P
	0.1 M	RT	Tetragonal HEWL crystals[m]	S	0.5 M NaCl	G	ΔB_{rel}	P
	0.1 M	100 K	Tetragonal HEWL crystals[m]	S	0.5 M NaCl	G	ΔB_{rel}	N

**Sodium Salicylate**	0.2 M	100 K	Azurin crystals[n]	S	5 mM NaAc pH 5.8,<br>1.85 M - 1.95 M $(NH4)_2SO_4$,<br>0.5 M $LiNO_3$	Sp	A632 nm	U
**Styrene**	0.002 M	RT	DOB immunoglobulin crystals[s,q]	C	70% 0.1M sodium borate, pH 8.4	G	I/I_0 for 2 or 3 reflections	P
	Saturated	100 K	Tetragonal HEWL crystals[o]	C	25 mM NaAc pH 4.5<br>0.5 M NaCl,<br>30% MPEG 5K<br>10 % *v/v* glycerol	Sp<br>G	$\|F_n\| - \|F_0\|$<br>Average I/I_0	N<br>N
	0.1 M	RT	Tetragonal HEWL crystals[m]	C	0.5 M NaCl	G	ΔB_{rel}	S
	0.1 M	100 K	Tetragonal HEWL crystals[m]	C	0.5 M NaCl	G	ΔB_{rel}	N
**Sucrose**	0.5 M	100 K	Disulphide/thiol model solutions[r]	N/A	0.1 M cystine,<br>0.5 M NaOH,<br>30% *v/v* EG, pH 8.97	Sp	A400 nm	N
***t*-Butanol**	0.5 M	100 K	Disulphide/thiol model solutions[r]	N/A	0.1 M cystine,<br>0.5 M NaOH,<br>30% *v/v* EG, pH 12.56	Sp	A400 nm	N
**TEMP**	0.1 M	100 K	Disulphide/thiol model solutions[r]	N/A	0.1 M cystine,<br>0.5 M NaOH,<br>30% *v/v* EG, pH 13.27	Sp	A400 nm	U
	0.5 M	RT	Tetragonal HEWL crystals[e]	S	0.1 M NaAc pH 4.5,<br>4-8% *w/v* NaCl	G	I/I_o	U

**Thiourea**	0.5 M	100 K	Disulphide/thiol model solutions[r]	N/A	0.1 M cystine, 0.25 M NaOH, 30% *v/v* EG, pH 10.87	Sp	A400 nm	U
	0.2 M	100 K	Azurin crystals[n]	S	5 mM NaAc pH 5.8, 1.85 M - 1.95 M $(NH4)_2SO_4$, 0.5 M $LiNO_3$	G	A632 nm	U
	0.4 M	RT, 100 K	Tetragonal HEWL crystals[m]	C	0.5 M NaCl	G	ΔB_{rel}	N
**Trehalose**	1 M	100K	Disulphide/thiol model solutions[r]	N/A	0.1 M cystine, 0.25 M NaOH, 30% *v/v* glycerol, pH 12.1	Sp	A400 nm	N
**Tris**	0.02 M	RT	β-galactosidase solutions[d]	N/A	0.01 M phosphate, pH 8.0	Sp	Mass of native polypeptide	P
	0.02 M	195 K	β-galactosidase solutions[d]	N/A	0.01 M phosphate, pH 8.0	Sp	Mass of native polypeptide	N
	0.02 M	RT	β-galactosidase lyophilised powders[d]	N/A	0.01 M phosphate, pH 8.0	Sp	Mass of native polypeptide	P
	0.02 M	195 K	β-galactosidase lyophilised powders[d]	N/A	0.01 M phosphate, pH 8.0	Sp	Mass of native polypeptide	N

[d] (Audette-Stuart *et al.*, 2005); [e] (Barker *et al.*, 2009); [f] (Betts, 2003); [g] (Borek *et al.*, 2007); [h] (Cascio *et al.*, 1984); [i] (De la Mora *et al.*, 2011); [j] (Holton, 2007); [k] (Jones *et al.*, 1987); l) (Kauffmann *et al.*, 2006); [m] (Kmetko *et al.*, 2011); [n] (Macedo *et al.*, 2009); [o] (Murray & Garman, 2002); [p] (Nowak *et al.*, 2009); [q] (Sarma & Zaloga, 1975); [r] (Southworth-Davies & Garman, 2007); [s] (Zaloga & Sarma, 1974).

References

Audette-Stuart, M., Houeé-levin, C. & Potier, M. (2005). *Radiation Physics and Chemistry* **72**, 301-306.

Barker, A. I., Southworth-Davies, R. J., Paithankar, K. S., Carmichael, I. & Garman, E. F. (2009). *Journal of Synchrotron Radiation* **16**, 205-216.

Betts, S. (2003).

Borek, D., Ginell, S. L., Cymborowski, M., Minor, W. & Otwinowski, Z. (2007). *Journal of Synchrotron Radiation* **14**, 24-33.

Cascio, D., Williams, R. & McPherson, A. (1984). *Journal of Applied Crystallography* **17**, 209-210.

De la Mora, E., Carmichael, I. & Garman, E. F. (2011). *Journal of Synchrotron Radiation* **18**, 346-357.

Holton, J. M. (2007). *Journal of Synchrotron Radiation* **14**, 51-72.

Jones, G. D. D., Lea, J. S., Symons, M. C. R. & Taiwo, F. A. (1987). *Nature* **330**, 772-773.

Kauffmann, B., Weiss, M. S., Lamzin, V. S. & Schmidt, A. (2006). *Structure* **14**, 1099-1105.

Kmetko, J., Warkentin, M. A., Englich, U. & Thorne, R. E. (2011). *Acta Crystallographica D* **67**, 881-893.

Macedo, S., Pechlaner, M., Schmid, W., Weik, M., Sato, K., Dennison, C. & Djinović-Carugo, K. (2009). *Journal of Synchrotron Radiation* **16**, 191-204.

Murray, J. & Garman, E. (2002). *Journal of Synchrotron Radiation* **9**, 347-354.

Nowak, E., Brzuszkiewicz, A., Dauter, M., Dauter, Z. & Rosenbaum, G. (2009). *Acta Crystallographica Section D: Biological Crystallography* **65**, 1004-1006.

Sarma, R. & Zaloga, G. (1975). *Journal of Molecular Biology* **98**, 479-484.

Southworth-Davies, R. J. & Garman, E. F. (2007). *Journal of Synchrotron Radiation* **14**, 73-83.

Zaloga, G. & Sarma, R. (1974). *Nature* **251**, 551-552.

Reference for software used for the computational chemistry calculations detailed of Section 2.3 of main paper:

Gaussian 09, Revision B, Frisch, M. J.; Trucks, G. W.; Schlegel, H. B.; Scuseria, G. E.; Robb, M. A.; Cheeseman, J. R.; Scalmani, G.; Barone, V.; Mennucci, B.; Petersson, G. A.; Nakatsuji, H.; Caricato, M.; Li, X.; Hratchian, H. P.; Izmaylov, A. F.; Bloino, J.; Zheng, G.; Sonnenberg, J. L.; Hada, M.; Ehara, M.; Toyota, K.; Fukuda, R.; Hasegawa, J.; Ishida, M.; Nakajima, T.; Honda, Y.; Kitao, O.; Nakai, H.; Vreven, T.; Montgomery, Jr., J. A.; Peralta, J. E.; Ogliaro, F.; Bearpark, M.; Heyd, J. J.; Brothers, E.; Kudin, K. N.; Staroverov, V. N.; Kobayashi, R.; Normand, J.; Raghavachari, K.; Rendell, A.; Burant, J. C.; Iyengar, S. S.; Tomasi, J.; Cossi, M.; Rega, N.; Millam, J. M.; Klene, M.; Knox, J. E.; Cross, J. B.; Bakken, V.; Adamo, C.; Jaramillo, J.; Gomperts, R.; Stratmann, R. E.; Yazyev, O.; Austin, A. J.; Cammi, R.; Pomelli, C.; Ochterski, J. W.; Martin, R. L.; Morokuma, K.; Zakrzewski, V. G.; Voth, G. A.; Salvador, P.; Dannenberg, J. J.; Dapprich, S.; Daniels, A. D.; Farkas, Ö.; Foresman, J. B.; Ortiz, J. V.; Cioslowski, J.; Fox, D. J. Gaussian, Inc., Wallingford CT, 2009
